# Adding Multimedia Animations to Exercise Therapy Provides No Additional Benefit for Rotator Cuff–Related Shoulder Pain: A Randomized Clinical Trial

**DOI:** 10.3390/jcm14227964

**Published:** 2025-11-10

**Authors:** Irene Pérez-Porta, Fernando García-Pérez, María Ángeles Pérez-Manzanero, María Alicia Urraca-Gesto, Aurora Araujo-Narváez, María Velasco-Arribas, Marcos José Navarro-Santana, Gustavo Plaza-Manzano, Elia Pérez-Fernández, Mariano Tomás Flórez-García

**Affiliations:** 1 Physical Therapy and Rehabilitation Unit, Hospital Universitario Fundación Alcorcón, 28922 Madrid, Spain; ipporta@salud.madrid.org (I.P.-P.); fernando.garcia.perez.1961@gmail.com (F.G.-P.); mpmanzanero@salud.madrid.org (M.Á.P.-M.); malicia.urraca@salud.madrid.org (M.A.U.-G.); auroramaria.araujo@salud.madrid.org (A.A.-N.); marianotomas.florez@salud.madrid.org (M.T.F.-G.); 2 International Doctoral School, Universidad Rey Juan Carlos, 28922 Alcorcón, Spain; 3 Department of Physical Therapy, Occupational Therapy Rehabilitation and Physical Medicine, Universidad Rey Juan Carlos, 28922 Alcorcón, Spain; 4 Research Unit, Hospital Universitario Fundación Alcorcón, 28922 Madrid, Spain; mvelascoa@salud.madrid.org (M.V.-A.); eliapf@salud.madrid.org (E.P.-F.); 5 Department of Medical Specialties and Public Health, Universidad Rey Juan Carlos, 28922 Alcorcón, Spain; 6 Department of Physical Therapy, Faculty of Nursing, Physiotherapy and Podiatry, Universidad Complutense de Madrid, 28040 Madrid, Spain; marconav@ucm.es; 7 Grupo InPhysio, Instituto de Investigación Sanitaria del Hospital Clínico San Carlos (IdISSC), 28040 Madrid, Spain

**Keywords:** physical therapy, shoulder, exercise, clinical trial, multimedia

## Abstract

**Background:** Exercise therapy is essential in managing rotator cuff-related shoulder pain. Multimedia tools may enhance adherence and engagement, but their added value over traditional materials remains uncertain. **Objective:** To compare an exercise program delivered through paper-based materials with or without addition of multimedia animations in individuals with rotator cuff-related shoulder pain. **Method:** A single-center open-label randomized clinical trial was conducted in [Blinded] between April 2023 and December 2024 Patients with rotator cuff-related shoulder pain were included. Both groups received seven face-to-face exercise sessions with a physical therapist and were randomized into receiving or not multimedia animations. The main outcome measure was Shoulder Pain and Disability Index at 6-week follow-up. Other outcomes were pain intensity (rest, during movement and at night), patients’ satisfaction, perceived improvement and expectations and patients’ adherence to the exercise program. Furthermore, patients’ perceived usability, usefulness and satisfaction with multimedia animations were also measured. Subjects were followed for 24 weeks. Adequate multilevel regression models were implemented. **Results:** A total of 154 subjects were included (80 in the control group and 74 in the experimental group). Both groups improved over time, but there were no significant between-group differences regarding Shoulder Pain and Disability Index, pain intensity, patients’ satisfaction, perceived improvement or expectations. Subjects showed a decrease in adherence to exercise over time, without significant between-group differences. **Conclusions:** The implementation of multimedia animations may not provide additional benefits when a well-designed paper-based program and therapist support are already established.

## 1. Introduction

Shoulder pain is one of the most common musculoskeletal complaints, with an estimated community prevalence of approximately 16% and incidence rates ranging from 7.7 to 62 per 1000 person-years [1]. Among its various etiologies, rotator cuff-related shoulder pain (RCRSP) is considered the most frequent diagnosis [2]. This umbrella term has been proposed to unify previously overlapping diagnostic categories, including subacromial impingement syndrome, subacromial pain syndrome, and rotator cuff tendinopathy [3]. The societal burden of shoulder pain is considerable. In Denmark, the average associated costs over a six-year period were EUR 11,334–EUR 25,711 [4], and in the United States, productivity losses related to absenteeism among individuals with partial or full-thickness rotator cuff pathology were estimated between USD 4382 and USD 5843 [5]. Furthermore, the incidence of RCRSP is rising among working-age adults and is more prevalent in rural communities [6], making accessible and effective management strategies essential.

Exercise therapy is recommended as first-line care for RCRSP and yields clinically meaningful improvements in pain and function [7]. Multiple delivery modes are used in practice, from standard printed instructions to multimedia or animation-based support, but evidence on whether enhanced delivery improves clinical outcomes remains inconsistent [8,9]. In addition, adherence is frequently linked with treatment response and may act as a mediator or moderator of clinical effects [10,11,12,13,14]. Accordingly, the specific gap we address is whether adding multimedia animations to a Consensus on Exercise Reporting Template (CERT)-aligned program improves pain and function compared with standard printed delivery and, additionally, whether it influences treatment adherence. Consequently, the primary hypothesis of our study was that adding multimedia animations would produce superior improvements in pain or function than printed materials alone

Therefore, the primary aim of this study was to compare the effects of a CERT-aligned supervised exercise program delivered with printed materials alone versus the same program supplemented with multimedia animations on shoulder pain-related disability in individuals with RCRSP. The secondary outcomes included pain intensity, expectation of improvement, satisfaction with treatment, global impression of improvement, perceived usability, usefulness and satisfaction of the multimedia animations and adherence to the home-exercise program.

## 2. Materials and Methods

### 2.1. Setting

This was a single center randomized clinical trial following the recommendations of the Consolidated Standards of Reporting Trials (CONSORT) (Supplementary Material S1) [15]. The study was conducted at Hospital Universitario Fundación Alcorcón (Madrid, Spain) between 7 April 2023 and 1 December 2024. The protocol of the study has been already published [16].

The study was approved by the ethics committee of Hospital Universitario Fundación Alcorcón and registered in ClinicalTrials (NCT05770908). This trial has received funding from the Instituto de Salud Carlos III and the European Union (PI19/01490). The funder had no influence on the study’s design, execution, analysis or publication of results. The study was conducted according to the Declaration of Helsinki. Patients were not involved in the design, conduct or reporting of the trial.

### 2.2. Participants

All participants signed an informed consent statement before participating in the study. The recruitment was conducted by three rehabilitative physicians, who were unaware of treatment allocation, from participants attending consult with non-traumatic shoulder pain. The inclusion and exclusion criteria were based on previous published literature and clinical knowledge [17]. Inclusion criteria were as follows [16]: adults between 18 and 80 years old with RCSP lasting for at least 3 months, diagnosed by means of pain equal or greater than 3/10 points on the numeric pain rating scale (NPRS) during active elevation, and met ≥1 clinical criteria with positive response to orthopedic tests (Neer, Hawkins–Kennedy and/or empty can). Exclusion criteria were as follows: history of trauma or signs of other shoulder pathologies such as instability, frozen shoulder or adhesive capsulitis, previous shoulder surgery, calcific tenonitis, severe arthrosis or neuralgic amyotrophy; presence of full-thickness rotator cuff tears; neck-related shoulder pain and/or radiculopathy or radicular pain; systemic disease (e.g., cancer, rhematic disorders, neurological disorders…); and severe psychiatric disorders.

### 2.3. Sample Size

The sample size was calculated using the ‘MBESS’ package of R software v.4.1.0, based on the precision of the adjusted mean difference from an analysis of covariance. The calculation was made for the Shoulder Pain and Disability Index (SPADI) at 3-month follow-up, assuming a standard deviation of 25 points, an allocation ratio of 1:1 and a correlation of 0.50 between baseline and 3-month follow-up. For a 95% confidence interval width of 15 points, the estimated sample size was 112 subjects, and assuming a 15% drop-out rate, the final sample size was composed of 132 subjects [16].

### 2.4. Interventions

All interventions were delivered by two physical therapists from Hospital Universitario Fundación Alcorcón with more than 5 years of experience in therapeutic exercise for shoulder disorders and trained together to standardize instruction and load progressions/regressions. All patients received a personalized exercise-and-education program with adjunct analgesics as needed. The content included shoulder exercises performed with elastic bands (Thera-Band^®^, 155 cm × 14.5 cm, yellow to silver for increasing resistance; The Hygenic Corporation, Akron, OH, USA), dumbbells (1–4 kg), and a small towel. Two criteria were used for progression/regression of exercise load: pain intensity and rate of perceived exertion (RPE). Patients were asked to work at an RPE ≥ 6/10 and to keep pain during exercise at or below 4/10, with symptoms returning to baseline within 2–3 h. If pain during or after exercise reached 5/10 or more, the load was reduced or levels were stepped back. The dose was set between 1 and 3 sets and 5–10 repetitions per exercise, with 1–2 min of rest between sets. Instructions were given to slowly achieve final position, hold it for 5 s and slowly return to the starting position. Frequency and duration were set at five individual in-person sessions of 30 min on alternating days over three weeks, followed by two in-person review sessions at 6 and 12 weeks to check the technique and adjust dosage. Sessions took place in the outpatient physical therapy unit with direct supervision during the seven hospital visits, encouraging patients to perform the taught exercises at home. At the beginning, the exercises were performed daily, and later in the program, the exercises were performed every other day. The comparator arm used “standard instruction,” meaning participants received verbal scripts and printed handouts with images. The experimental arm received the same content plus access to self-explanatory multimedia animations. To support fidelity and adherence, therapists used a brief session checklist, delivered standardized cues, and reviewed a patient self-reported calendar of home sessions. Patients also recorded weekly pain to guide progression. Full details, including the completed CERT checklist [16], are provided in the Supplementary Material S2.

**Figure 1:**
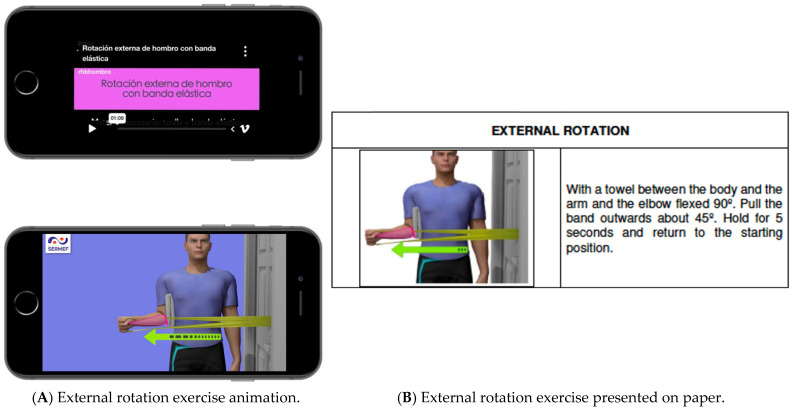
Example exercises. (**A**) External rotation with multimedia animation. (**B**) External rotation in printed format.

Patients were randomized to receive either printed exercise handouts alone (pictures with explanatory text) or the same printed handouts plus access to a webpage (http://rhbhombro.com/) with multimedia animations (self-explanatory video demonstrations) of the prescribed exercises (Figure 1) [16]. Verbal instructions were identical in both groups. As an example, for external rotation with an elastic band, participants received the following directions: “You need an elastic band and a towel to perform this exercise. The elastic band must be attached to a door handle, and you must stand next to it. The elbow should be in 90° of flexion forming a right angle, holding the towel against the body. To perform the exercise, pull the elastic band outwards by about 45° of external rotation, making it taut without dropping the towel. The rest of the body should not move during the performance of the exercise. Hold this position for 5 s and slowly return to the starting position.”

### 2.5. Randomization and Blinding

The randomization procedure was conducted with a 1:1 allocation ratio using Epidat software v.4.2 (Xunta de Galicia, Galicia, Spain) by an external statistician. Allocation concealment was achieved using sequentially numbered opaque envelopes.

The rehabilitative physicians that were responsible for recruitment were blinded to treatment allocation, whereas the physical therapists administering the interventions and collecting outcome measures, as well as the patients, were aware of the assignments, making this an open-label study.

### 2.6. Outcomes

All measurements were conducted in Hospital Universitario Fundación Alcorcón by three rehabilitative physicians and two physical therapists. The main outcome measure was collected at baseline, 6-week and 12- and 24-week follow-ups after finishing treatment. Full details about outcomes and measurement schedules are available in the published protocol [16].

The main outcome was shoulder pain-related disability, measured with the SPADI questionnaire, which was transculturally adapted into the Spanish language in 2015 [18].

Pain intensity was assessed with the numeric pain rating scale (NPRS) [19], patients’ expectations and satisfaction were evaluated using an 11-point ordinal rating scale and patients’ perceived improvement was measured using the Patient Global Impression of Improvement (PGI-I) scale.

Furthermore, patients’ perceptions on the usability of the multimedia animations were assessed using the System Usability Scale (SUS) [20] and patients’ perceived usefulness and satisfaction of multimedia animations were evaluated using a 5-point Likert-type scale.

Finally, exercise adherence was assessed using self-fillable calendars completed by the patients, where they recorded the days when they performed the exercises in each week.

### 2.7. Statistical Analyses

All statistical analyses were conducted using R software v.4.1.0 (R Core Team 2021). For the description of quantitative variables, the mean, standard deviation (SD), median, first and third quartiles and minimum and maximum values were reported. For categorical variables, absolute frequencies and percentages were reported.

The analysis of between-group differences in NPRS measures and SPADI were conducted using generalized least squares models fitted by restricted maximum likelihood. The baseline measurement was included as a covariate, and the time factor (6, 12, 24) was modeled using a linear spline. Finally, an autoregressive-moving average lag 1 (AR1) correlation structure was assumed. Post hoc pairwise comparisons were conducted with a t-Student test with Bonferroni’s correction, to obtain adjusted mean differences. For the analysis of adherence to the exercise program, a similar analysis was performed but including adherence at 3-week follow-up as the covariate of the model and using a restricted cubic spline with three knots to model the time variable. The residual plots and variograms of the model were also reported.

The analysis of between-group differences in patients’ expectations was conducted using a generalized least squares model with a compound-symmetry correlation structure, since there are only two post-treatment measurements (time 3 and 6). On the other hand, the analysis of between-group differences in patients’ satisfaction was conducted using a *t*-Student test.

A logistic regression model was conducted to analyze the relationship between baseline variables and missingness. For dealing with missing data, a multiple imputation procedure was used (five imputations) by means of predictive mean matching procedure with the ‘mice’ R package v.3.18.0.

Finally, the analysis of between-group differences in patients’ perceived improvement was conducted using a rank-based mixed Analysis of Variance (ANOVA), following the method of Brunner, Domhof and Langer of 2002.

All analyses were conducted as intention-to-treat analyses, and assuming an alpha level of 0.05 with 95% confidence intervals (CI).

## 3. Results

**Figure 2:**
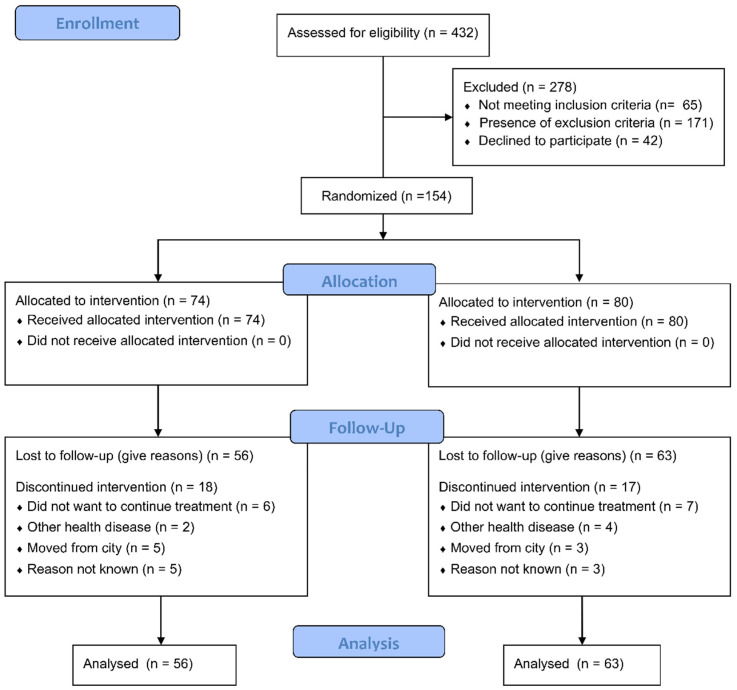
CONSORT flow diagram.

The final sample included 154 subjects (Figure 2); 80 received the control intervention (female, 63.7%; mean age, 55.28 years), and 74 received the experimental intervention (female, 54.1%; mean age, 54.63). The summary of descriptive statistics of demographic data is presented in Table 1.

The logistic regression analyses revealed no association between baseline variables and missingness in any outcome measure (Supplementary Material S3).

### 3.1. Between-Group Differences in Shoulder Pain and Disability Outcomes

**Figure 3:**
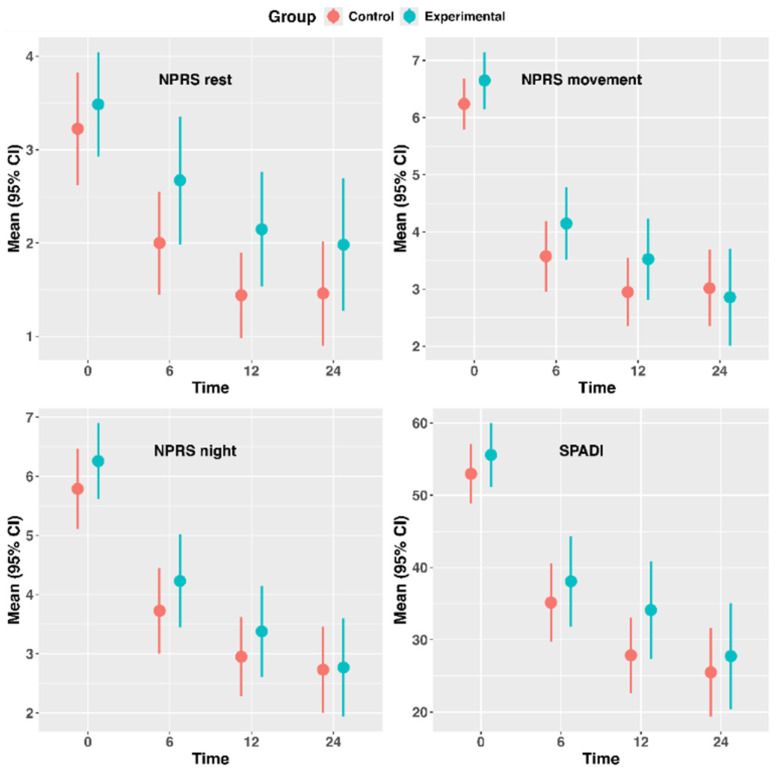
Error bar plot for shoulder pain and disability outcome measures. Dots are estimated means, and vertical lines represent the 95% confidence interval of the mean. Abbreviations: NPRS, numeric pain rating scale; SPADI, Shoulder Pain and Disability Index.

The descriptive statistics of the outcome measures are presented in Table 2, and the error bar plots in Figure 3. Both groups improved over time in NPRS at rest (c^2^(df = 4) = 10.25, *p* = 0.036), during movement (c^2^(df = 4) = 16.35, *p* = 0.003) and at night (c^2^(df = 4) = 23.61, *p* < 0.001).

There was also a significant effect of time for SPADI in both groups (c^2^(df = 4) = 27.93, *p* < 0.001). However, there were no significant main effects for group factor, nor significant time-by-group interactions for any outcome measure (Supplementary Material S4), meaning that there were no significant adjusted mean differences between-groups at any follow-up, neither in full available data nor in the imputed dataset (Table 3). The residual plots and variograms of the models are presented in Supplementary Material S5 and the histograms of outcome measures in Supplementary Material S6.

### 3.2. Between-Group Differences in Perceived Improvement, Satisfaction and Expectations with Received Treatment and Adherence to the Exercise Program

The rank-based approach for the analysis of perceived improvement revealed a significant main effect for time (W(df = 2) = 35.26, *p* < 0.001), but not for group factor (W(df = 1) = 0.02, *p* = 0.90). Furthermore, there was a non-significant time-by-group interaction (W(df = 2) = 2.59, *p* = 0.27). The descriptive statistics are presented in Supplementary Material S4, showing a trend towards greater perceived improvement over time in both groups. Regarding patients’ expectations, there were no significant main effects for time and group factors, and there was a non-significant time-by-group interaction (Supplementary Material S4). Furthermore, there were no significant differences in patients’ satisfaction at 6-week follow-up (mean difference: −0.15; 95% CI, −0.90 to 0.60) or 24-week follow-up (mean difference: −0.08; 95% CI, −0.91 to 0.75). Finally, the analysis of adherence to the exercise program revealed a significant main effect for time (c^2^(df = 4) = 50.30, *p* < 0.001) but not for group factor (c^2^(df = 3) = 3.74, *p* = 0.29), and there was a non-significant time-by-group interaction (c^2^(df = 4) = 0.07, *p* = 0.97). The descriptive statistics for patients’ expectations, satisfaction and adherence are presented in Supplementary Material S4.

### 3.3. Web-Based Application Perceptions

Descriptive data regarding SUS, utility and satisfaction about the web-based application within the subjects in the experimental group at 12-week follow-up is presented in Supplementary Material S4. In summary, subjects showed a mean rating in SUS of 78.55 and median utility and satisfaction scores of 3 over a maximum of 4 points.

## 4. Discussion

This single-center open-label randomized clinical trial evaluated the effects of the addition of multimedia animations to traditional paper-based exercises in subjects with RCRSP. Overall, no between-group differences were found in shoulder disability, pain intensity (at rest, during movement, or at night), patients’ expectations, satisfaction and perceived improvement or adherence to the exercise program. However, patients reported high levels of usability, usefulness and satisfaction with the multimedia animations.

Our results showed that both exercise interventions led to improvements in shoulder-related disability and pain intensity. The benefits of exercise for patients with RCRSP have been extensively documented, with consistent evidence supporting its effectiveness in reducing pain intensity and improving disability and function [7,21]. However, important uncertainties remain regarding the optimal type, intensity, progression and delivery methods of exercise interventions [22]. Exercise is not a one-size-fits-all intervention, but rather a therapeutic modality that must be individualized to each person in order to be effective [22,23]. Furthermore, it is also a patient-dependent intervention, as its success relies on whether the patient actually performs the prescribed exercises [11]. Patients’ adherence to the exercise program is dependent on their expectations with treatment, their ability to learn and perform the exercises, their self-efficacy strategies and their time and equipment availability, among others 7.

For these reasons, some researchers started to investigate strategies to improve patients’ adherence, including the use of videos or multimedia animations, aiming to facilitate exercise learning and enhance self-efficacy through the use of technologies that are more engaging than a simple paper-based format [8,9]. However, evidence on this topic in shoulder pain populations remains scarce [24,25].

To the best of the authors’ knowledge, this is the first randomized clinical trial to evaluate the effectiveness of adding multimedia animations to paper-based exercises in individuals with RCRSP. Contrary to our initial hypothesis, there were no differences in any outcome measure between both groups, meaning that the use of multimedia animations provided no additional benefit over the traditional paper-based method.

These findings suggest that the clinical benefit of therapeutic exercise may lie more in the appropriateness and personalization of the program itself rather than in the format in which it is delivered. Previously published literature has also shown that the use of multimedia approaches to exercise instruction does not seem to provide additional benefits regarding patient-related outcomes [9].

Several factors may account for these findings. First, it may be a ceiling effect (usually seen in bounded outcome measures), whereby the face-to-face sessions, simplicity of the prescribed exercises and well-structured paper-based materials were already sufficient to achieve optimal outcomes.

Second, the therapeutic alliance fostered by the expertise of the two physical therapists delivering the intervention, along with the repeated face-to-face sessions and the simplicity of the prescribed exercises, may have reduced the potential additive value of the multimedia animations, by enhancing patients’ engagement, highlighting the importance of clinician guidance in promoting adherence and performance [26]. Furthermore, most of the participants were middle-aged adults with primary or secondary education, which may have facilitated their ability to understand the exercises through paper-based explanations alone [27]. There are some studies that have evaluated the effectiveness and feasibility of telerehabilitation in comparison to non-telerehabilitation treatment for subjects with RCRSP [28,29]. Recently, Timurtaş et al. [30] published a randomized clinical trial evaluating the effectiveness of a mobile application versus synchronous videoconferencing in individuals with RCRSP. They found that the videoconferencing group improved more than the mobile application group, suggesting that some form of synchronous guidance may be necessary when treating patients with shoulder pain. Overall, despite new technologies potentially supporting patients in performing their prescribed exercises, the therapist’s presence still seems to play a more crucial role [26]. Future studies should explore whether multimedia tools might be more beneficial in younger or more tech-savvy populations, or in contexts where face-to-face guidance is limited or unavailable.

Finally, adherence was relatively high in both groups, possibly due to the simplicity of the exercises and the use of tracking calendars, which may have limited the observable added value of adherence-enhancing tools like animations. Substantial benefits from exercise programs can be achieved even without high levels of adherence, and greater adherence does not necessarily lead to greater improvements in pain and function [9]. For that reason, adherence in this study may have reached the minimum threshold required to produce therapeutic benefits, making the addition of multimedia animations not better in terms of pain and function outcomes.

### Limitations

The present study has some limitations that should be considered. First, it was an open-label trial where therapists knew the treatment allocation. Second, there was a mid-term follow-up of the patients, so the results cannot be generalized to long-term follow-up. Finally, the exercise protocol was limited to a predefined set of exercises, which may limit the generalizability of the findings to other types of exercise programs.

Additionally, participants received five face-to-face sessions with experienced physical therapists, which may have contributed ceiling effects and limited generalizability to settings with less therapist contact. Future studies should compare exercise-and-education protocols that are otherwise similar but delivered with fewer face-to-face sessions (or alternative delivery modes) to assess the impact of therapist-contact dose on clinical outcomes.

## 5. Conclusions

The addition of multimedia animations to a paper-based exercise program seems to add no benefits regarding improvements in shoulder disability, pain, patients’ satisfaction, perceived improvement or adherence in subjects with RCRSP. Given the lack of added clinical benefit, the implementation of multimedia animations may not provide additional benefits when a well-designed paper-based program and therapist support are already established.

## Figures and Tables

**Table 1:** Demographic data of included participants.

Group	Control (*n* = 80)			Experimental (*n* = 74)		
Variable	N	Mean	SD	N	Mean	SD
Age, years	80	55.3	11.1	74	54.6	12.9
BMI	80	26.9	4.5	72	26.4	4.4
Sex, Male	29	36.2%		34	45.9%	
Education						
Primary	26	32.5%		25	33.8%	
Secondary	21	26.3%		27	36.5%	
No education	1	1.3%				
University	32	40.0%		22	29.7%	
Pain side						
Left	25	31.3%		28	37.8%	
Right	55	68.8%		46	62.2%	
Pain side equal dominant side	53	66.3%		46	62.2%	
Previous PT	3	3.8%		6	8.1%	
Previous analgesic	34	42.5%		42	56.8%	

Abbreviations: SD, standard deviation; PT, physical therapy.

**Table 2:** Descriptive statistics of shoulder pain and disability outcome measures.

Group	Time	*n*	Mean	SD	Median	P25	P75	Min.	Max.
NPRS, rest									
Control	Baseline	80	3.23	2.70	3	0.75	5	0	9
	6 wk	73	2	2.37	1	0	3	0	8
	12 wk	59	1.44	1.75	1	0	2	0	6
	24 wk	63	1.46	2.22	1	0	2	0	10
Experimental	Baseline	74	3.49	2.42	4	2	5	0	10
	6 wk	61	2.67	2.67	1	0	5	0	9
	12 wk	61	2.15	2.40	1	0	3	0	9
	24 wk	56	1.98	2.65	1	0	3	0	9
NPRS, movement									
Control	Baseline	80	6.24	1.99	6	5	8	0	10
	6 wk	73	3.58	2.64	3	2	5	0	10
	12 wk	59	2.95	2.30	3	1	4.5	0	9
	24 wk	63	3.02	2.66	2	1	4.5	0	10
Experimental	Baseline	74	6.65	2.15	7	5	8	0	10
	6 wk	61	4.15	2.48	4	3	6	0	9
	12 wk	61	3.52	2.77	3	1	6	0	9
	24 wk	56	2.86	3.15	2	0	5.25	0	10
NPRS, night									
Control	Baseline	80	5.79	3.06	6	3	8	0	10
	6 wk	73	3.72	3.08	3	1	7	0	10
	12 wk	59	2.95	2.57	2	1	5	0	8
	24 wk	63	2.73	2.88	2	0	4	0	10
Experimental	Baseline	74	6.26	2.77	7	5	8	0	10
	6 wk	61	4.23	3.07	4	2	7	0	10
	12 wk	61	3.38	3.01	3	1	6	0	10
	24 wk	56	2.77	3.11	1.5	0	5.25	0	9
SPADI									
Control	Baseline	80	52.96	18.62	51.57	40.58	67.11	9.23	89.17
	6 wk	73	35.13	23.09	29.23	16.15	58.46	0	92.5
	12 wk	59	27.84	20.10	27.69	10.39	43.59	0	86.92
	24 wk	63	25.48	24.37	20.77	5.39	34.62	0	96.92
Experimental	Baseline	74	55.58	18.99	59.23	43.65	68.08	16.92	90.78
	6 wk	61	38.08	24.29	36.15	16.15	61.67	0	84.62
	12 wk	60	34.10	26.13	27.69	12.07	58.08	0	87.69
	24 wk	56	27.73	27.47	18.08	3.66	47.69	0	90
Patient’s expectations									
Control	Baseline	78	8.83	1.45	9	8	10	2	10
	3 wk	74	8.81	1.58	9	8	10	2	10
	6 wk	69	8.54	1.93	9	8	10	2	10
Experimental	Baseline	72	8.65	1.91	9	8	10	1	10
	3 wk	66	8.15	1.99	9	8	10	2	10
	6 wk	59	8.19	2.51	9	8	10	0	10
Patient’s satisfaction									
Control	6 wk	69	7.91	1.96	8	7	9	2	10
	24 wk	53	7.89	1.94	8	7	10	3	10
Experimental	6 wk	59	7.76	2.35	8	7	10	0	10
	24 wk	51	7.80	2.32	9	6	10	1	10

Abbreviations: SD, standard deviation; P., percentile; Min., minimum; Max., maximum; NPRS, numeric pain rating scale; SPADI, Shoulder Pain and Disability Index.; wk, weeks.

**Table 3:** Adjusted between-group differences in shoulder pain and disability outcomes, patients’ expectations and satisfaction with received treatment.

Time	Original Dataset			Imputed Dataset		
	Adjusted Between-Group Mean Difference *	Lower 95% CI	Upper 95% CI	Adjusted Between-Group Mean Difference *	Lower 95% CI	Upper 95% CI
NPRS, rest						
6 wk	−0.49	−1.25	0.27	−0.47	−1.25	0.31
12 wk	−0.17	−0.95	0.61	−0.29	−1.02	0.45
24 wk	−0.37	−1.18	0.43	−0.38	−1.23	0.46
NPRS, movement						
6 wk	−0.46	−1.35	0.43	−0.32	−1.18	0.54
12 wk	−0.17	−1.08	0.74	−0.18	−1.04	0.67
24 wk	0.30	−0.64	1.23	0.16	−0.84	1.15
NPRS, night						
6 wk	−0.27	−1.22	0.67	−0.28	−1.33	0.77
12 wk	0.17	−0.80	1.14	0.01	−1.00	1.02
24 wk	0.27	−0.73	1.26	0.15	−0.90	1.20
SPADI						
6 wk	−0.74	−8.25	6.77	−0.86	−8.48	6.76
12 wk	−1.54	−9.22	6.14	−1.72	−8.84	5.41
24 wk	−0.29	−8.16	7.57	−0.54	−9.02	7.93
Patients’ expectations						
3 wk	0.53	−0.05	1.11	0.52	−0.07	1.10
6 wk	0.20	−0.40	0.80	0.45	−0.13	1.04
Patients’ satisfaction with received treatment						
6 wk	0.08	−0.66	0.81	0.37	−0.38	1.13
24 wk	−0.03	−0.84	0.78	−0.06	−0.80	0.67

* Mean differences are adjusted by setting baseline measurement at the median, and calculated as the experimental minus control group. Abbreviations: CI, confidence interval; NPRS, numeric pain rating scale.; SPADI, Shoulder Pain and Disability Index; wk, weeks.

## Data Availability

Data are available upon request. The data that support the findings of this study are available from the first author.

## Supplementary Materials

The following supporting information can be downloaded at https://www.mdpi.com/article/10.3390/jcm14227964/s1, Supplementary Material S1: Consort Checklist; Supplementary Material S2: Description of exercise intervention; Supplementary Material S3: Missing data analyses; Supplementary Material S4: Supplementary data for outcome measure analyses; Supplementary Material S5: Residual plots and variograms for generalized least squares regression models of outcome measures; Supplementary Material S6: Histograms of outcome measures.

## Abbreviations

The following abbreviations are used in this manuscript:

CERT	Consensus on Exercise Reporting Template
NPRS	Numeric Pain Rating Scale
PGI-I	Patient Global Impression of Improvement
SPADI	Shoulder Pain and Disability Index
SUS	System Usability Scale
RCRSP	Rotator Cuff-Related Shoulder Pain
