# Supplementary material for: Adding Multimedia Animations to Exercise Therapy Provides No Additional Benefit for Rotator Cuff–Related Shoulder Pain: A Randomized Clinical Trial
Source: J Clin Med. 2025 Nov 10;14(22):7964. doi: 10.3390/jcm14227964 (PMC12653082; doi:10.3390/jcm14227964)
Supplement: Supplementary file 1 [file jcm-14-07964-s001.zip › jcm-3882616-supplementary.pdf]

SUPPLEMENTARY MATERIAL 1

CONSORT checklist

Section/topic	No	CONSORT 2025 checklist item description	Reported on page no.
**Title and abstract**			
Title and structured abstract	1a	Identification as a randomised trial	1
	1b	Structured summary of the trial design, methods, results, and conclusions	2
**Open science**			
Trial registration	2	Name of trial registry, identifying number (with URL) and date of registration	5
Protocol and statistical analysis plan	3	Where the trial protocol and statistical analysis plan can be accessed	5
Data sharing	4	Where and how the individual de-identified participant data (including data dictionary), statistical code and any other materials can be accessed	5
Funding and conflicts of interest	5a	Sources of funding and other support (eg, supply of drugs), and role of funders in the design, conduct, analysis and reporting of the trial	5
	5b	Financial and other conflicts of interest of the manuscript authors	20
**Introduction**			
Background and rationale	6	Scientific background and rationale	4-5
Objectives	7	Specific objectives related to benefits and harms	5
**Methods**			
Patient and public involvement	8	Details of patient or public involvement in the design, conduct and reporting of the trial	5-6
Trial design	9	Description of trial design including type of trial (eg, parallel group, crossover), allocation ratio, and framework (eg, superiority, equivalence, non-inferiority, exploratory)	5-7
Changes to trial protocol	10	Important changes to the trial after it commenced including any outcomes or analyses that were not prespecified, with reason	5
Trial setting	11	Settings (eg, community, hospital) and locations (eg, countries, sites) where the trial was conducted	5
Eligibility criteria	12a	Eligibility criteria for participants	6
	12b	If applicable, eligibility criteria for sites and for individuals delivering the interventions (eg, surgeons, physiotherapists)	5-6
Intervention and comparator	13	Intervention and comparator with sufficient details to allow replication. If relevant, where additional materials describing the intervention and comparator (eg, intervention manual) can be accessed	6-7
Outcomes	14	Prespecified primary and secondary outcomes, including the specific measurement variable (eg, systolic blood pressure), analysis metric (eg, change from baseline, final value, time to event), method of aggregation (eg, median, proportion), and time point for each outcome	7-8
Harms	15	How harms were defined and assessed (eg, systematically, non-systematically)	7-8
Sample size	16a	How sample size was determined, including all assumptions supporting the sample size calculation	6
	16b	Explanation of any interim analyses and stopping guidelines	6
Randomisation:			7
Sequence generation	17a	Who generated the random allocation sequence and the method used	
	17b	Type of randomisation and details of any restriction (eg, stratification, blocking and block size)	7

			Reported on page no.
Allocation concealment mechanism	18	Mechanism used to implement the random allocation sequence (eg, central computer/telephone; sequentially numbered, opaque, sealed containers), describing any steps to conceal the sequence until interventions were assigned	7
Implementation	19	Whether the personnel who enrolled and those who assigned participants to the interventions had access to the random allocation sequence	7
Blinding	20a	Who was blinded after assignment to interventions (eg, participants, care providers, outcome assessors, data analysts)	7
	20b	If blinded, how blinding was achieved and description of the similarity of interventions	7
Statistical methods	21a	Statistical methods used to compare groups for primary and secondary outcomes, including harms	8-9
	21b	Definition of who is included in each analysis (eg, all randomised participants), and in which group	8-9
	21c	How missing data were handled in the analysis	8-9
	21d	Methods for any additional analyses (eg, subgroup and sensitivity analyses), distinguishing prespecified from post hoc	8-9
**Results**			
Participant flow, including flow diagram	22a	For each group, the numbers of participants who were randomly assigned, received intended intervention, and were analysed for the primary outcome	10
	22b	For each group, losses and exclusions after randomisation, together with reasons	10
Recruitment	23a	Dates defining the periods of recruitment and follow-up for outcomes of benefits and harms	7 and 10-12
	23b	If relevant, why the trial ended or was stopped	NA
Intervention and comparator delivery	24a	Intervention and comparator as they were actually administered (eg, where appropriate, who delivered the intervention/comparator, how participants adhered, whether they were delivered as intended (fidelity))	10
	24b	Concomitant care received during the trial for each group	11
Baseline data	25	A table showing baseline demographic and clinical characteristics for each group	10-11, Table 1
Numbers analysed, outcomes and estimation	26	For each primary and secondary outcome, by group:<br>● the number of participants included in the analysis<br>● the number of participants with available data at the outcome time point<br>● result for each group, and the estimated effect size and its precision (such as 95% confidence interval)<br>● for binary outcomes, presentation of both absolute and relative effect size	10-11, Table 1-3, Suplemental material 2-5
Harms	27	All harms or unintended events in each group	NA
Ancillary analyses	28	Any other analyses performed, including subgroup and sensitivity analyses, distinguishing pre-specified from post hoc	NA
**Discussion**			
Interpretation	29	Interpretation consistent with results, balancing benefits and harms, and considering other relevant evidence	12-14
Limitations	30	Trial limitations, addressing sources of potential bias, imprecision, generalisability, and, if relevant, multiplicity of analyses	14

Citation: Hopewell S, Chan AW, Collins GS, Hróbjartsson A, Moher D, Schulz KF, et al. CONSORT 2025 Statement: updated guideline for reporting randomised trials. BMJ. 2025; 388:e081123. https://dx.doi.org/10.1136/bmj-2024-081123
© 2025 Hopewell et al. This is an Open Access article distributed under the terms of the Creative Commons Attribution License (https://creativecommons.org/licenses/by/4.0/), which permits unrestricted use, distribution, and reproduction in any medium, provided the original work is properly cited.

*We strongly recommend reading this statement in conjunction with the CONSORT 2025 Explanation and Elaboration and/or the CONSORT 2025 Expanded Checklist for important clarifications on all the items. We also recommend reading relevant CONSORT extensions. See www.consort-spirit.org.

SUPPLEMENTARY MATERIAL 2

Description of exercise intervention

DETAILED DESCRIPTION OF THE EXERCISE INTERVENTION

Six exercise programs were created based on the information provided above. The programs 1 (basic), 2 (basic plus scapular and internal rotation), and 3 (basic plus scapular) are aimed at patients no mobility limitation (strengthening exercises only). On the other hand, the programs 4 (scapular and stretching), 5 (basic plus scapular and stretching), and 6 (complete) are aimed at patients with limited mobility. The exercises were identical in the experimental and control groups. The multimedia animations are available at: http://rhbhombro.com/. The exercises included within each program are presented in the following table:

Exercises	Program					
	1	2	3	4	5	6
Horizontal row		X	X	X	X	X
Supine scapular protraction		X	X	X	X	X
Scaption	X	X	X		X	X
External rotation	X	X	X		X	X
Internal rotation		X				X
Posterior capsule stretching				X	X	X

The included exercises are as follows:

HORIZONTAL ROW	
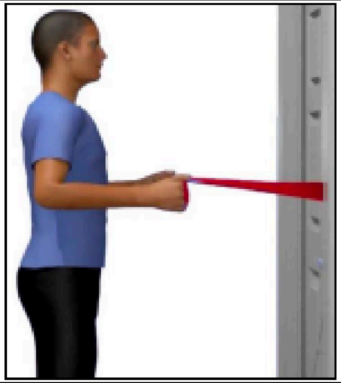	Arms with elbows bent at 90º. Pull the band with your hands making it tense, bringing the elbows and hands backwards, bringing the shoulder blades together. Hold for 5 seconds and return to starting position.

SUPINE SCAPULAR PROTRACTION	
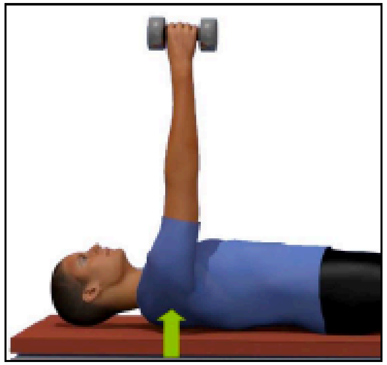	Shoulder elevated at 90º, and elbow extended holding a weight with the hand. Raise the weight while keeping the arm vertical. Hold the position for 5 seconds and return to the starting position.
**SCAPTION**	
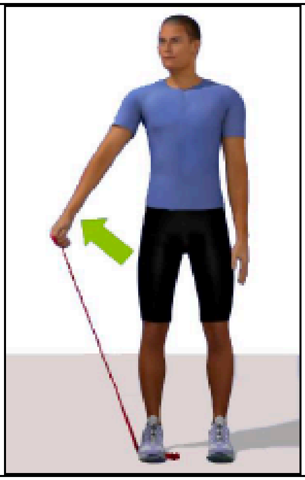	Slowly separate the arm upwards pulling the band without getting the arm horizontal. Hold for 5 seconds and return to the starting position.
**EXTERNAL ROTATION**	
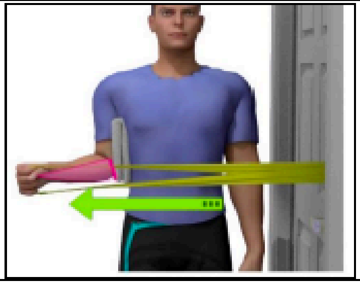	With a towel between the body and the arm and the elbow flexed 90º. Pull the band outwards about 45º. Hold for 5 seconds and return to the starting position.

**INTERNAL ROTATION**	
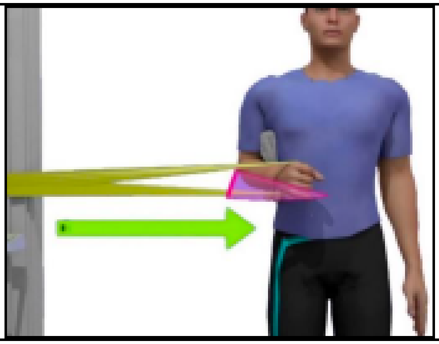	With a towel between the body and the arm and the elbow flexed 90º. Pull the band inward about 45º. Hold for 5 seconds and return to the starting position.
**POSTERIOR CAPSULE STRETCHING**	
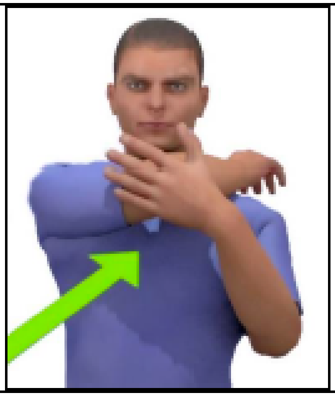	Perform a 90º flexion of the shoulder and place the hand of the affected side over the healthy shoulder. With the other hand push the elbow backwards.

Exercises description and verbal instructions

The detailed description (verbal instructions) of each exercise that was provided to the patient is as follows:

HORIZONTAL ROW

- You need an elastic band to perform this exercise.
- The starting position is standing/sitting in front of a closed door, with an
- elastic band hooked to the door handle.
- The arms should be about 45° away from the trunk and the elbows are
- kept bent at 90°.
- The band and forearms should be parallel to the floor.
- The spine must be kept straight during the performance of the exercise.
- To perform the exercise, pull the elastic band with your hands making it
- taut, bringing the elbows and hands backwards, bringing the shoulder
- blades together.
- Hold this position for 5 seconds and slowly return to the starting position.

SUPINE SCAPULAR PROTRACTION

- You need a dumbbell to perform this exercise.
- The starting position is lying on the floor face up. If you are more
- comfortable, you can place a cushion under your head.
- The arm with which the exercise is going to be performed remains
- perpendicular to the floor, with the elbow stretched out, while holding a
- dumbbell in your hand.
- The spine shouldn’t be twisted during the performance of the exercise.
- To perform the exercise, take your shoulder off the floor by bringing your
- arm upwards, holding the weight towards the ceiling.
- Hold this position for 5 seconds and slowly return to the starting position.

SCAPTION

- You need an elastic band to perform this exercise.
- The starting position is standing facing forward with legs slightly apart,
- arms straight and relaxed along the body.
- One end of the band should be stepped on with the foot, and the other

- grasped with the hand of the symptomatic arm.
- The band should be slightly taut.
- The spine must be kept straight during the performance of the exercise.
- To perform the exercise the entire arm should be slowly pulled upward
- by pulling the band up to 30-40 degrees of elevation in the scapular
- plane.
- During the performance of the exercise, the elbow should be kept
- straight, the body shouldn't be rotated, and the shoulder shouldn't be
- shrugged.
- Hold this position for 5 seconds and slowly return to the starting position

EXTERNAL ROTATION

- You need an elastic band and a towel to perform this exercise.
- The elastic band is attached to a door handle, and you must stand next
- to it.
- The elbow should be in 90º flexion forming a right angle, holding the
- towel between the elbow and the body.
- To perform the exercise, pull the elastic band outwards by about 45º of
- external rotation, making it taut without dropping the towel.
- The rest of the body should not move during the performance of the
- exercise.
- Hold this position for 5 seconds and slowly return to the starting position.

INTERNAL ROTATION

- You need an elastic band and a towel to perform this exercise.
- The elastic band is attached to a door handle, and you must stand next
- to it.
- The elbow should be in 90º flexion forming a right angle, holding the
- towel between the elbow and the body.
- To perform the exercise, pull the elastic band inward by about 45º of
- internal rotation, making it taut without dropping the towel.
- The rest of the body should not move during the performance of the
- exercise.

- Hold this position for 5 seconds and slowly return to the starting position.

POSTERIOR CAPSULE STRETCHING

- The starting position is standing.
- The palm of the hand of the side to be stretched is placed on top of the
- other shoulder, and the hand of the side that is to assist the stretch is
- placed resting on the opposite elbow.
- To perform the exercise, direct the elbow toward the opposite shoulder
- while your hand slides lightly down the back of the shoulder. Try to
- increase the movement by pushing slowly with the other hand on the
- elbow, without rotating the trunk.
- Hold this position for 20 seconds and slowly return to the starting

position.

Consensus on Exercise Reporting Template (CERT)

Item 1: Detailed description of the type of exercise equipment

The needed equipment to perform the exercises are:

- Elastic bands (Thera-Band®) with a length of 155cm and a width of 14.5cm. There are six type of bands which, in order from least to most resistant, are as follows: yellow, red, green, blue, black, and silver.1
- Dumbbells with varying weights from 1kg to 4kg.
- Small size towel.

Item 2: Detailed descriptions of the qualifications, teaching/supervising expertise and/or training undertaken by the exercise instructor

The exercise instructors will be two physical therapists working at the Hospital Universitario Fundación Alcorcón. They have 4 to 30 years of experience treating patients with musculoskeletal shoulder disorders using therapeutic exercise. All the therapists were provided with a teaching session for the instruction on the exercise program, in aim to standardize the explanations given to the patients, as well as criteria for load progression and regression.

Item 3: Describe whether the exercises are performed individually or in a group

All sessions that patients will receive at the hospital will be performed individually with a 30-minute duration. The patients will attend 5 sessions every other day, and two revision sessions, one at 1-month, and another one at 3 month-followup.

Item 4: Describe whether exercises are supervised or unsupervised and how they are delivered.

The abovementioned seven exercise sessions will be supervised at the hospital with a physical therapist. However, the patient will be asked to perform the trained exercise at home all days until three-month follow-up. After that, the patient will be encouraged to keep up with the exercise at least 3-days per week until last follow-up with the medical doctor at 6-month follow-up. During the supervised sessions, the physical therapist will observe the exercise performance, and correct any compensations made by the patient, ensuring an adequate pattern of movement. Furthermore, the dosage will be modified according to patients' characteristics at each session.

Item 5: Detailed description of how adherence to exercise is measured and reported.

Adherence to the exercise program will be measured using a self-reported calendar, in which the patient should mark the days he/she will perform the exercises. Furthermore, patient's will be asked to rate their pain intensity within last week on Sundays.

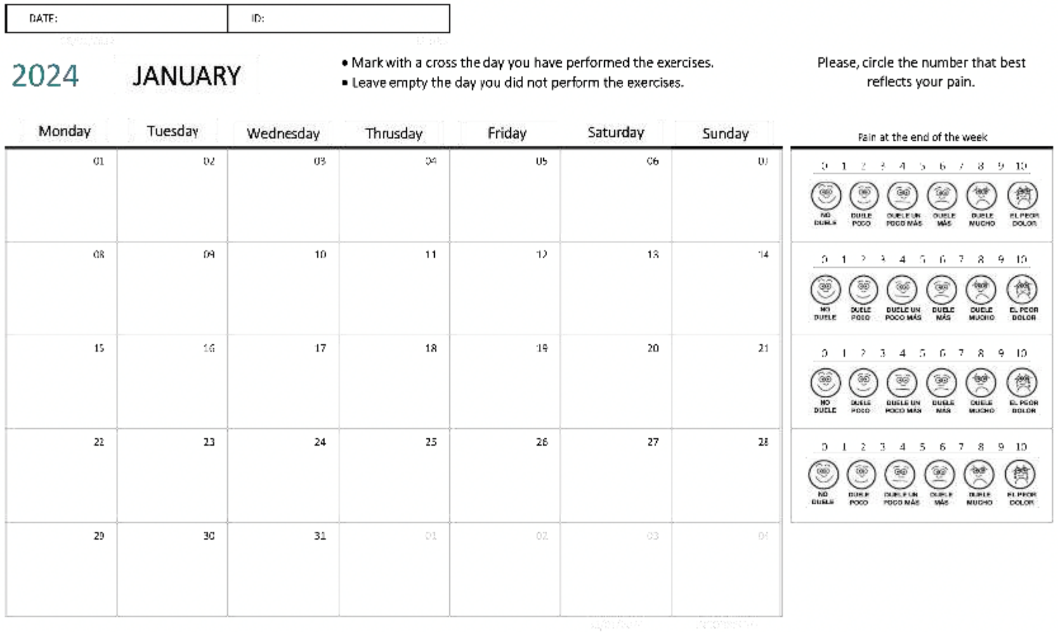

Item 6: Detailed description of the motivation strategies.

In order to motivate the patient to perform the exercises, information will be provided throughout the treatment sessions about his or her pathology and the importance of exercise in his or her recovery. In addition, the physiotherapists will give positive feedback in the face-to-face sessions, with motivational messages, placing greater emphasis on the points well performed by the patients within each exercise and their progress in tolerance to the load.

Item 7(a): Detailed description of the rule(s) for determining exercise progression.

Two criteria were used for progression/regression of exercise load: pain intensity and perceived sensation of exertion.

The intensity of pain should be mild during the exercises (i.e., ≤ 4/10 in a verbal numeric pain rating scale). Furthermore, although there may be a small increase in pain with exercise, it should return to baseline within 2 to 3 hours after exercise. In addition, the patient should feel a sensation of moderate effort when performing the exercises, with a perceived exertion value equal or greater than 6 in a 0-10 verbal rating scale.

The first criterion to consider is pain intensity, followed by perceived sensation of exertion. If the patient does not have moderate pain within the exercise, and has low perceived exertion, the load will be increased. If, after that, the pain increases, he/she would be asked to return to the initial load. The algorithm of guidance provided to the physiotherapists for the loading profession is presented as follows.

SUPPLEMENTARY MATERIAL 3

Missing data analyses

Missing data map for experimental group

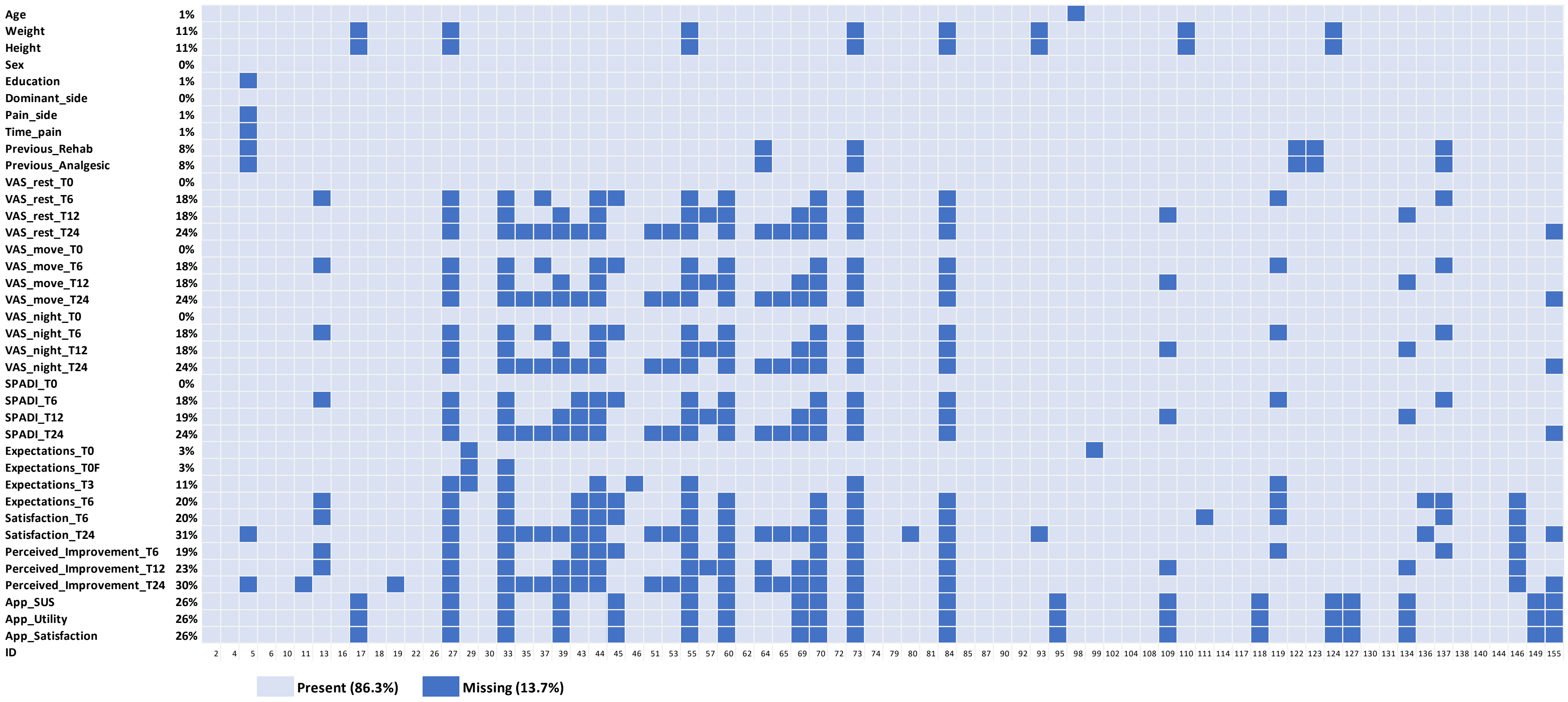

Missing data map for control group

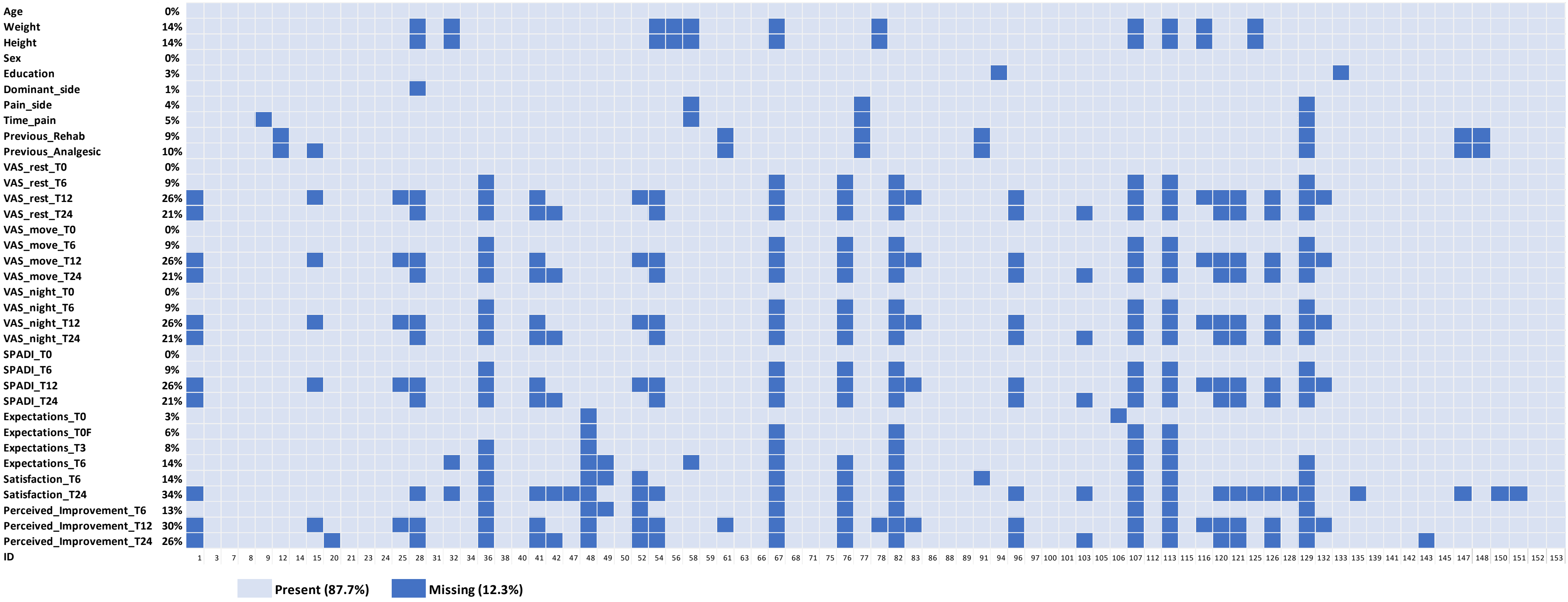

Logistic regression model for relationship between baseline variables and missing outcome data at any follow-up in experimental group

```
Logistic Regression Model

 lrm(formula = formula_lrm, data = DF_Irene_Exp)


                    Model Likelihood     Discrimination    Rank Discrim.
                          Ratio Test            Indexes          Indexes
 Obs          31    LR chi2     19.71     R2       1.000    C       1.000
  0           28    d.f.           23     R2(23,31)0.000    Dxy     1.000
  1            3    Pr(> chi2) 0.6595    R2(23,8.1)0.000    gamma   1.000
 max |deriv| 0.4                          Brier    0.000    tau-a   0.181

                        Coef     S.E.      Wald Z Pr(>|Z|)
 Intercept              -53.2330 1252.8391 -0.04  0.9661
 Age                     -0.5334    8.9875 -0.06  0.9527
 Weight                   0.2067   14.3698  0.01  0.9885
 Height                   0.2155    6.3343  0.03  0.9729
 Sex=Male                 1.0271  152.7345  0.01  0.9946
 Education=Secondary      7.9490  248.8610  0.03  0.9745
 Education=University    11.6362  529.8556  0.02  0.9825
 Dominant_side=Right     17.5787  421.6985  0.04  0.9667
 Pain_side=Left          20.9550  281.3369  0.07  0.9406
 Pain_side=Right         23.8853  291.2775  0.08  0.9346
 Previous_Rehab=Yes       5.0552  462.9201  0.01  0.9913
 Previous_Analgesic=Yes  -1.8492  138.3742 -0.01  0.9893
 VAS_rest_T0              0.9427   29.4934  0.03  0.9745
 VAS_move_T0              1.0403   48.1697  0.02  0.9828
 VAS_night_T0            -1.6989   42.4313 -0.04  0.9681
 SPADI_T0                 0.2129    4.8224  0.04  0.9648
 Expectations_T0          0.7829   61.5827  0.01  0.9899
 Expectations_T0F         2.0002   92.0831  0.02  0.9827
 Expectations_T3         -0.9110  218.5975  0.00  0.9967
 Satisfaction_T6         -1.7264  104.7413 -0.02  0.9868
 Satisfaction_T24        -0.1278   51.4131  0.00  0.9980
 App_SUS                 -0.6527   11.9342 -0.05  0.9564
 App_Utility              2.9213  132.5360  0.02  0.9824
 App_Satisfaction         1.7612  165.1027  0.01  0.9915
```

Logistic regression model for relationship between baseline variables and missing outcome data at any follow-up in control group

```
Logistic Regression Model

 lrm(formula = formula_lrm_cont, data = DF_Irene_Cont)

                      Model Likelihood      Discrimination    Rank Discrim.
                            Ratio Test             Indexes          Indexes
 Obs            39    LR chi2     29.87      R2       1.000    C       1.000
  0             34    d.f.           19      R2(19,39)0.243    Dxy     1.000
  1              5    Pr(> chi2) 0.0535    R2(19,13.1)0.564    gamma   1.000
 max |deriv| 0.07                            Brier    0.000    tau-a   0.229

                        Coef      S.E.      Wald Z Pr(>|Z|)
 Intercept              -154.0026 2715.6867 -0.06  0.9548
 Age                       0.8260   12.7971  0.06  0.9485
 Weight                   -1.0481   13.6246 -0.08  0.9387
 Height                    0.8382   17.4404  0.05  0.9617
 Sex=Male                  3.6173  263.0883  0.01  0.9890
 Education=Secondary     -26.8831  344.4987 -0.08  0.9378
 Education=University      1.9851  202.2894  0.01  0.9922
 Dominant_side=Right      27.0559  695.6712  0.04  0.9690
 Pain_side=Right         -11.6211  290.7484 -0.04  0.9681
 Previous_Rehab=Yes      -13.6791  522.9933 -0.03  0.9791
 Previous_Analgesic=Yes  -13.0875  202.0914 -0.06  0.9484
 VAS_rest_T0               5.1585   94.1984  0.05  0.9563
 VAS_move_T0              -0.9273   56.7415 -0.02  0.9870
 VAS_night_T0             -0.9481   42.2703 -0.02  0.9821
 SPADI_T0                  0.1485    9.8838  0.02  0.9880
 Expectations_T0         -10.1930  106.1744 -0.10  0.9235
 Expectations_T0F         17.2225  166.9295  0.10  0.9178
 Expectations_T3          -8.8240  121.7046 -0.07  0.9422
 Satisfaction_T6           7.5383   83.4169  0.09  0.9280
 Satisfaction_T24         -4.5185   69.0457 -0.07  0.9478
```

SUPPLEMENTARY MATERIAL 4

Supplementary data for outcome measures analyses

Supplementary Table S1. Main effects and time-by-group interactions for the generalized regression models implemented.

Variable	Main effect time	Main effect group	Time-by-group interaction
NPRS, rest	χ^2(df=4) = 10.25<br>p = .036	χ^2(df=3) = 2.51<br>p = .47	χ^2(df=2) = 1.07<br>p = .58
NPRS, movement	χ^2(df=4) = 16.35<br>p = .003	χ^2(df=3) = 2.14<br>p = .54	χ^2(df=2) = 2.06<br>p = .36
NPRS, night	χ^2(df=4) = 23.61<br>p < .001	χ^2(df=3) = 1.48<br>p = .69	χ^2(df=2) = 1.47<br>p = .48
SPADI	χ^2(df=4) = 27.93<br>p < .001	χ^2(df=3) = 0.20<br>p = .98	χ^2(df=2) = 0.16<br>p = .92
Expectations	χ^2(df=2) = 1.48<br>p = .48	χ^2(df=2) = 3.21<br>p = .20	χ^2(df=1) = 1.01<br>p = .32
Adherence	χ^2(df=4) = 50.30<br>p < .001	χ^2(df=3) = 3.74<br>p = .29	χ^2(df=2) = 0.07<br>p = .97

Abbreviations: NPRS, numeric pain rating scale; SPADI, Shoulder Pain and Disability Index; df, degrees of freedom.

Supplementary Table S2. Descriptive statistics of perceived improvement over time.

Variable	N	Percent	N	Percent
Group	Control		Experimental	
Perceived Improvement, T6	70		60	
... Much worst	1	1.4%	0	0%
... Something worst	3	4.3%	0	0%
... No change	6	8.6%	8	13.3%
... Something better	35	50%	32	53.3%
... Much better	25	35.7%	19	31.7%
... Totally recovered	0	0%	1	1.7%
Perceived Improvement, T12	56		57	
... Much worst	0	0%	1	1.8%
... Something worst	0	0%	2	3.5%
... No change	4	7.1%	9	15.8%
... Something better	20	35.7%	18	31.6%
... Much better	28	50%	24	42.1%
... Totally recovered	4	7.1%	3	5.3%
Perceived Improvement, T24	59		52	
... Much worst	2	3.4%	0	0%
... Something worst	2	3.4%	1	1.9%
... No change	3	5.1%	4	7.7%
... Something better	15	25.4%	11	21.2%
... Much better	29	49.2%	25	48.1%
... Totally recovered	8	13.6%	11	21.2%

Supplementary Table S3. Descriptive statistics about perceptions on the use of the web-based application**.**

Variable	N	Mean	SD	Min	Pctl. 25	Median	Pctl. 75	Max
SUS	55	78.545	17.231	45	65	80	95	100
Utility	55	2.818	1.203	0	2	3	4	4
Satisfaction	55	3.091	1.159	0	3	3	4	4

Abbreviations: SD, standard deviation, Pctl., percentile, Min, minimum; Max, maximum; SUS, System Usability Scale.

Supplementary Table S4. Descriptive statistics about adherence within the control group.

Time	n	Mean	SD	Median	Pctl. 25	Pctl. 75	Min	Max
0	68	4.25	2.01	5	3	6	0	7
1	61	5.79	1.55	7	5	7	2	7
2	59	5.08	1.95	5	4	7	0	7
3	56	5.08	2.08	6	4	7	0	7
4	54	4.81	2.34	6	3	7	0	7
5	55	4.70	2.08	5	4	7	0	7
6	53	4.45	1.97	4	3	6	0	7
7	51	4.43	2.21	5	3	6	0	7
8	52	4.38	2.21	5	3	6	0	7
9	52	4.44	2.13	4	3	6	0	7
10	52	4.61	1.98	5	3	6.25	0	7
11	53	4.43	1.90	4	3	6	0	7
12	53	4.11	2.15	4	2	6	0	7
13	51	4.09	2.19	5	2	6	0	7
14	47	3.91	2.03	4	3	5.5	0	7
15	42	4.19	2.15	5	3	6	0	7
16	41	4.39	2.06	4	4	6	0	7
17	39	4.28	2.01	4	3	6	0	7
18	36	4.27	1.68	4	3	5	1	7
19	35	4.45	1.83	4	3	6	1	7
20	35	3.85	1.95	4	3	5	0	7
21	34	4.32	2.02	5	3	6	0	7
22	33	3.81	2.24	4	3	5	0	7
23	32	3.34	2.07	3.5	2	4	0	7

Abbreviations: SD, standard deviation, Pctl., percentile, Min, minimum; Max, maximum; SUS, System Usability Scale.

Supplementary Table S5. Descriptive statistics about adherence within the experimental group.

Time	n	Mean	SD	Median	Pctl. 25	Pctl. 75	Min	Max
0	64	4.42	2.12	5	3.75	6	0	7
1	58	6.06	1.41	7	6	7	0	7
2	57	5.61	1.80	6	5	7	0	7
3	55	5.58	2.06	7	4.5	7	0	7
4	54	5.29	1.97	6	4	7	0	7
5	54	5.22	1.81	5	4.25	7	0	7
6	55	4.83	2.17	5	3	7	0	7
7	54	4.35	2.44	5	3	6.75	0	7
8	52	4.96	2.19	6	4	7	0	7
9	50	4.84	2.05	5	4	6	0	7
10	48	4.66	2.52	6	3.75	7	0	7
11	49	4.53	2.41	5	3	7	0	7
12	47	4.95	2.21	6	4	7	0	7
13	44	4.81	2.23	5	3	7	0	7
14	39	4.61	2.20	5	3	7	0	7
15	38	4.68	2.24	5	3	7	0	7
16	37	4.64	2.31	5	3	7	0	7
17	34	4.88	2.25	6	3.25	7	0	7
18	34	4.11	2.71	4.5	2	7	0	7
19	34	4.44	2.50	5	3	7	0	7
20	33	4.60	2.23	5	3	7	0	7
21	33	4.60	2.14	5	3	7	0	7
22	33	4.09	2.76	4	2	7	0	7
23	31	4.12	2.80	5	1.5	7	0	7

Abbreviations: SD, standard deviation, Pctl., percentile, Min, minimum; Max, maximum; SUS, System Usability Scale.

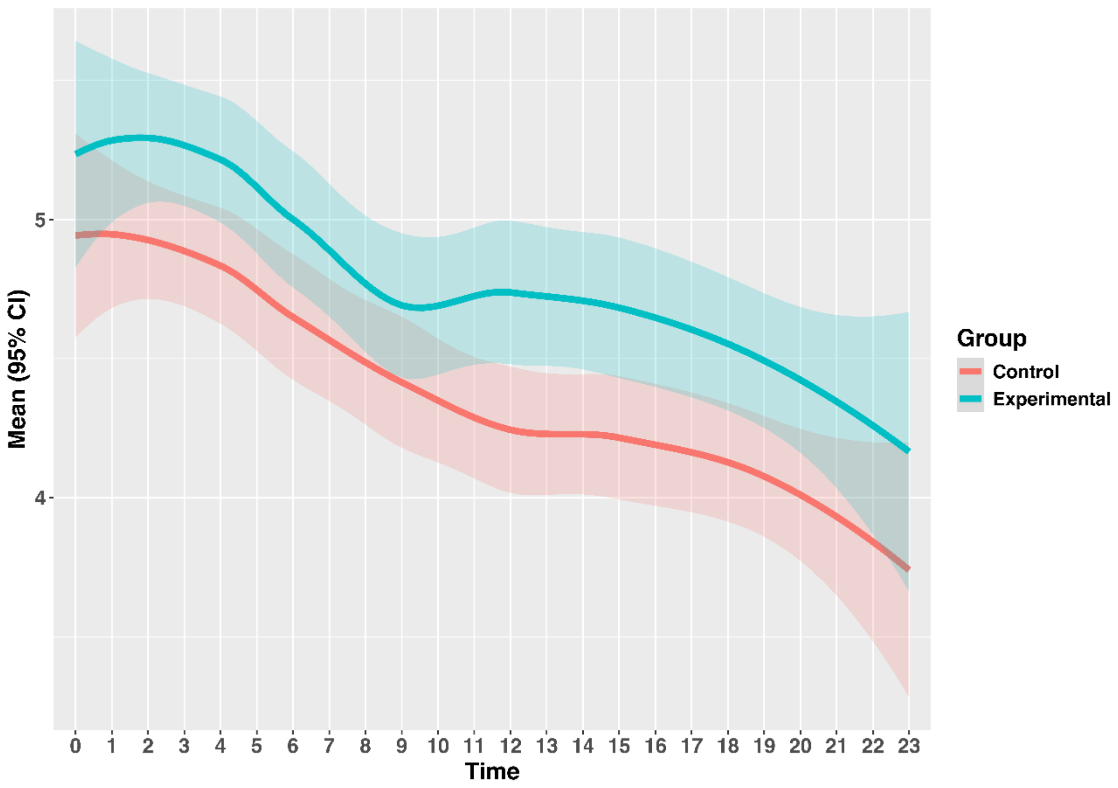

Supplementary Figure S1. LOESS plot for patients' adherence to the exercise program.

SUPPLEMENTARY MATERIAL 5

Residual plots and variograms for generalized least squares regression models of outcome measures

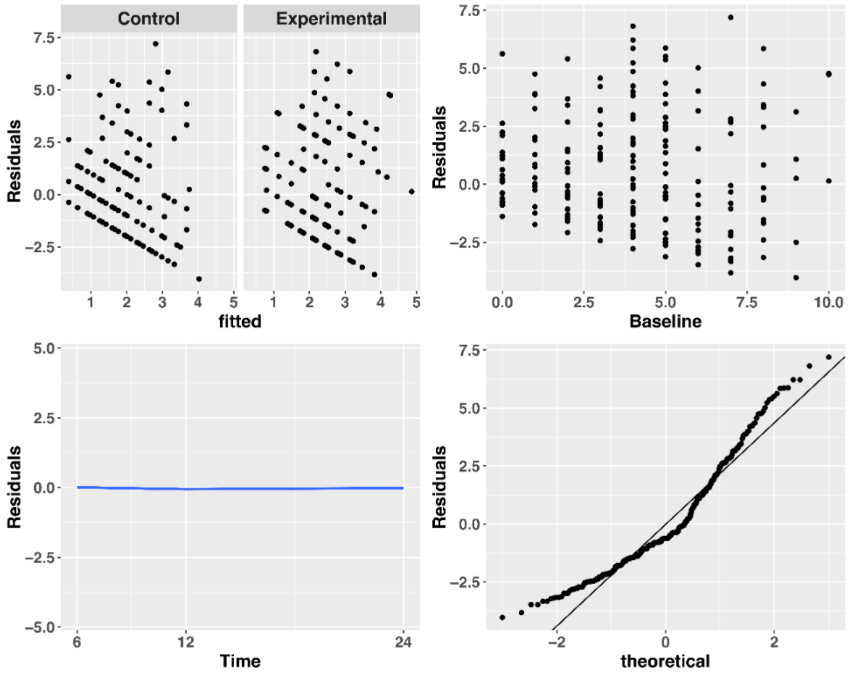

Figure S1. *Residual plots for generalized least squares model of numeric pain rating scale at rest.*

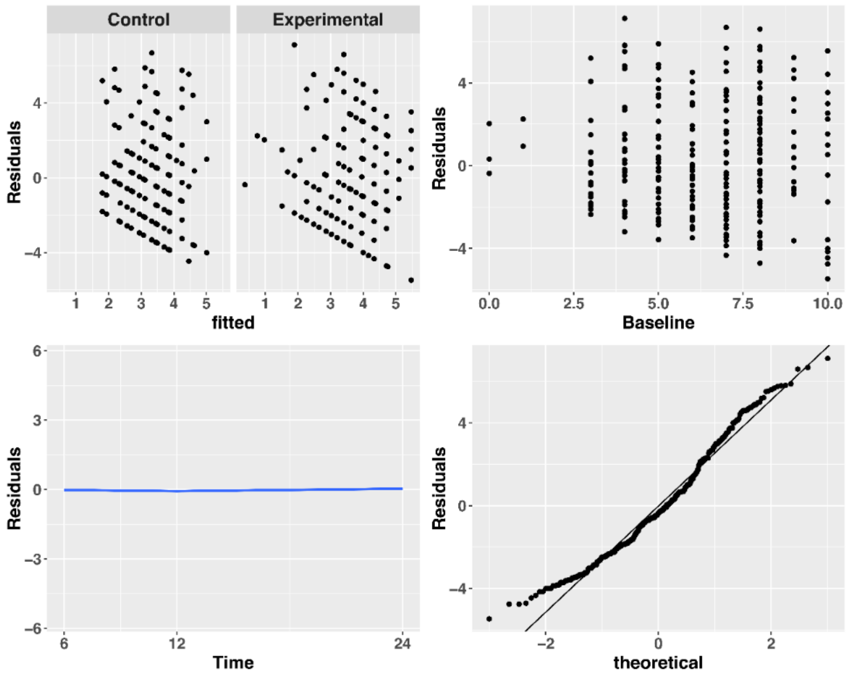

Figure S2. *Residual plots for generalized least squares model of numeric pain rating scale during movement.*

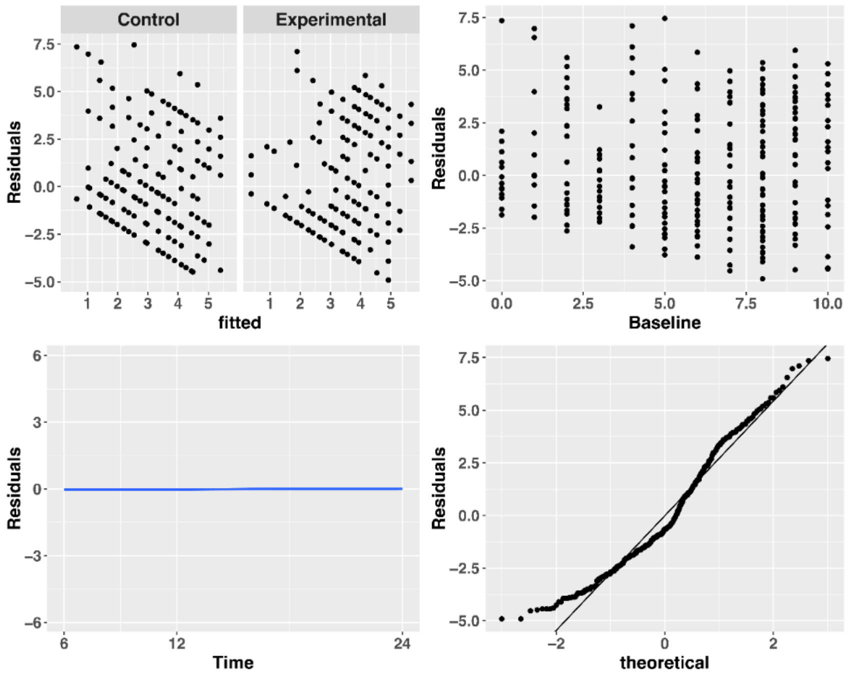

Figure S3. *Residual plots for generalized least squares model of numeric pain rating scale at night.*

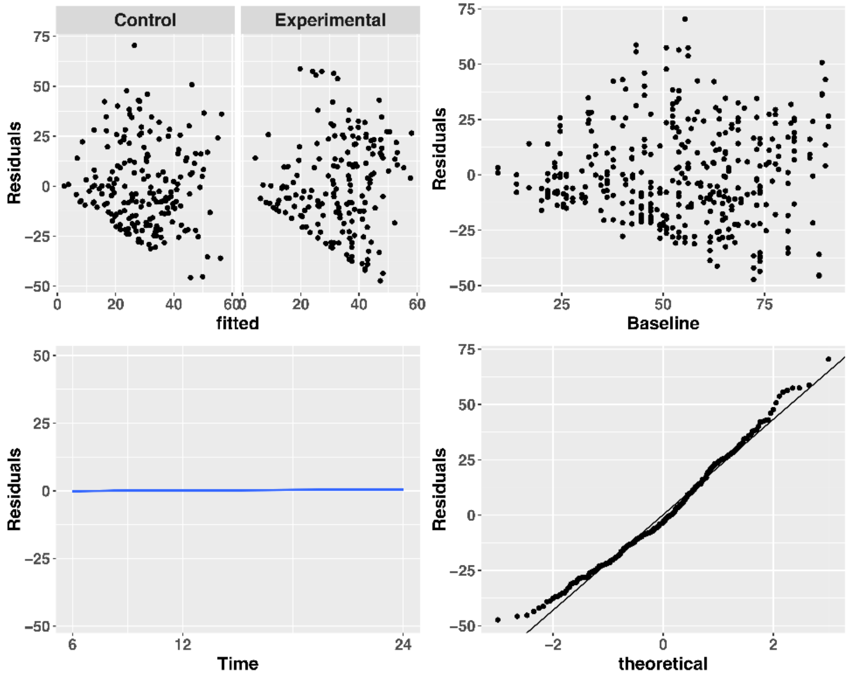

Figure S4. *Residual plots for generalized least squares model of Shoulder Pain and Disability Index.*

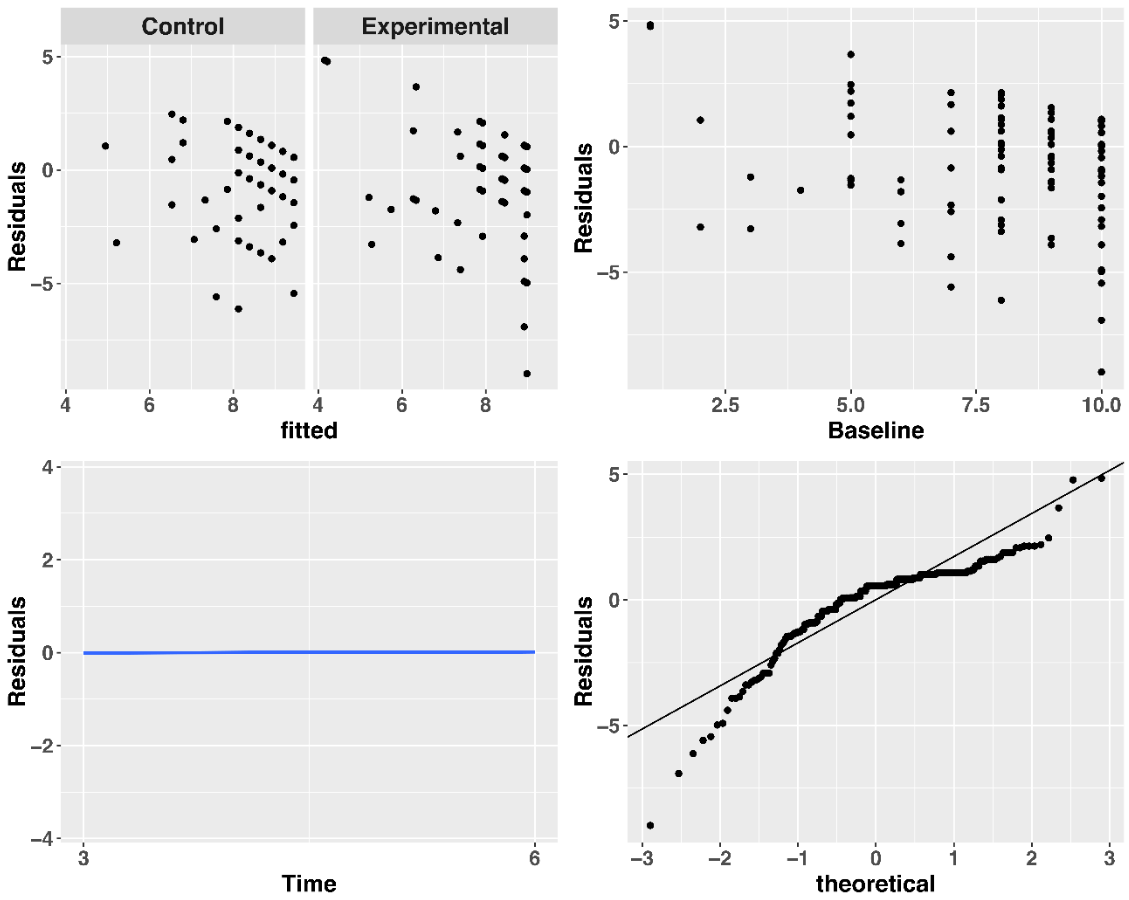

Figure S5. *Residual plots for generalized least squares model of patients' expectations.*

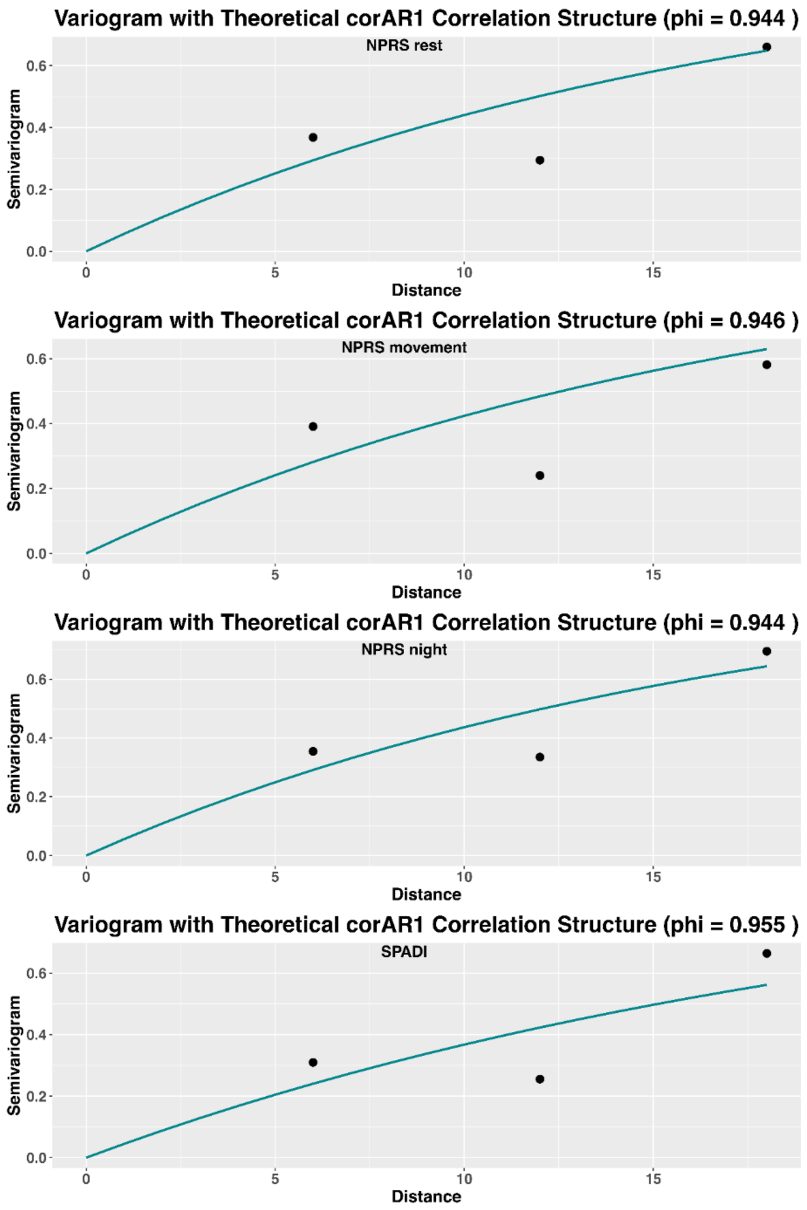

Figure S6. *Variograms for generalized least squares models. Abbreviations: NPRS, numeric pain rating scale; SPADI, Shoulder Pain and Disability Index.*

SUPPLEMENTARY MATERIAL 6

Histograms of outcome measures

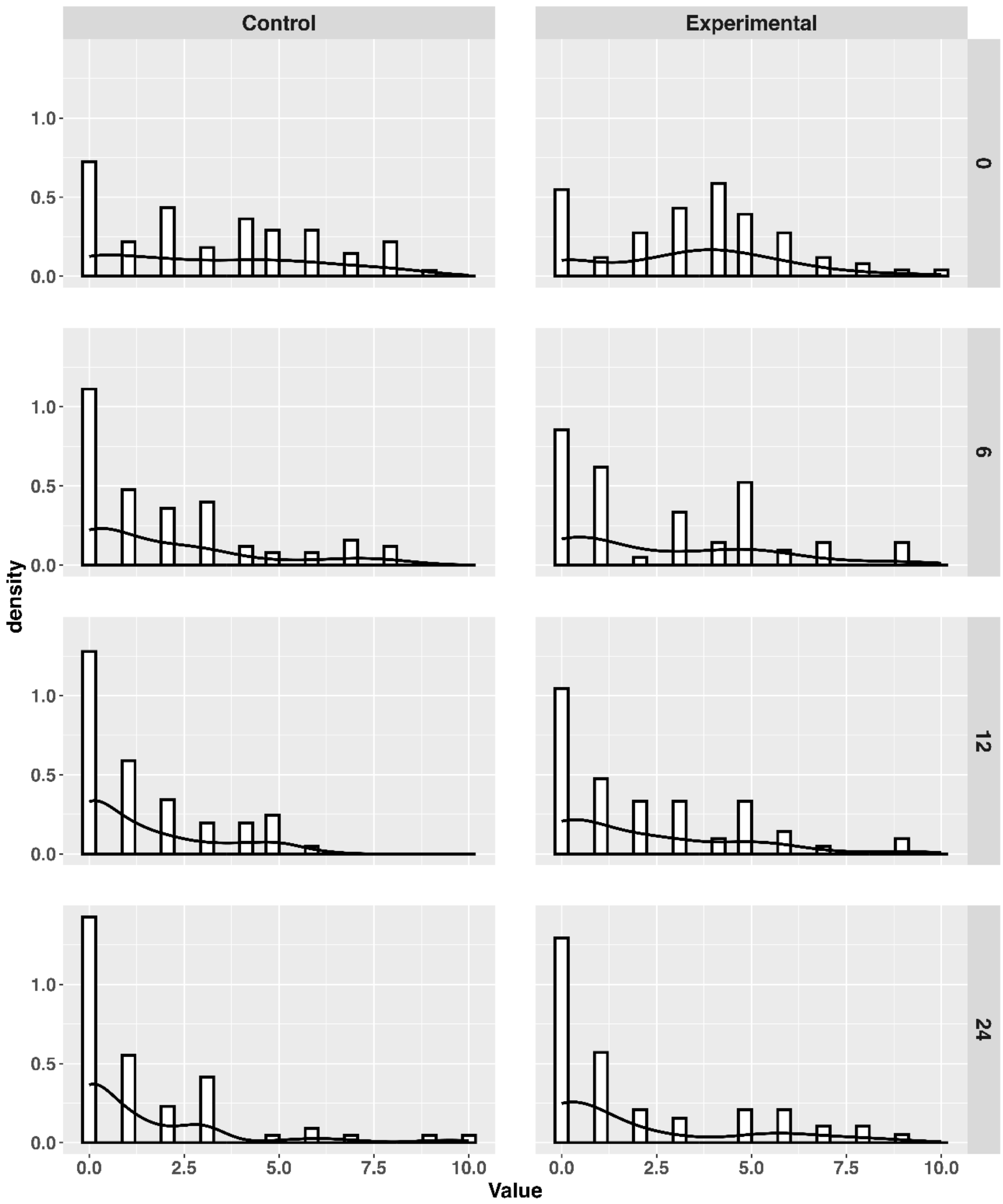

Figure S1. *Histograms of numeric pain rating scale at rest.*

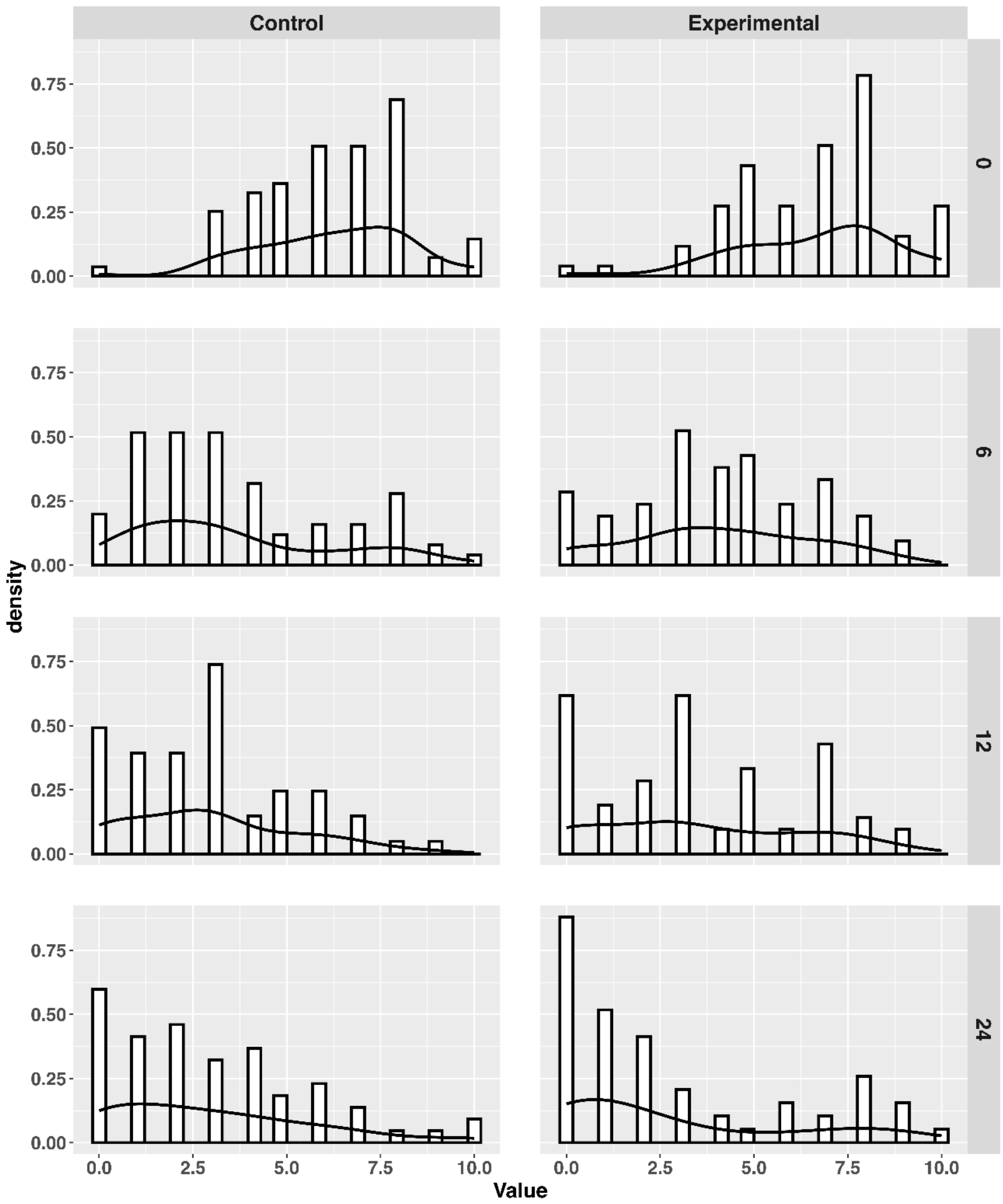

FigureS 2. *Histograms of numeric pain rating scale during movement.*

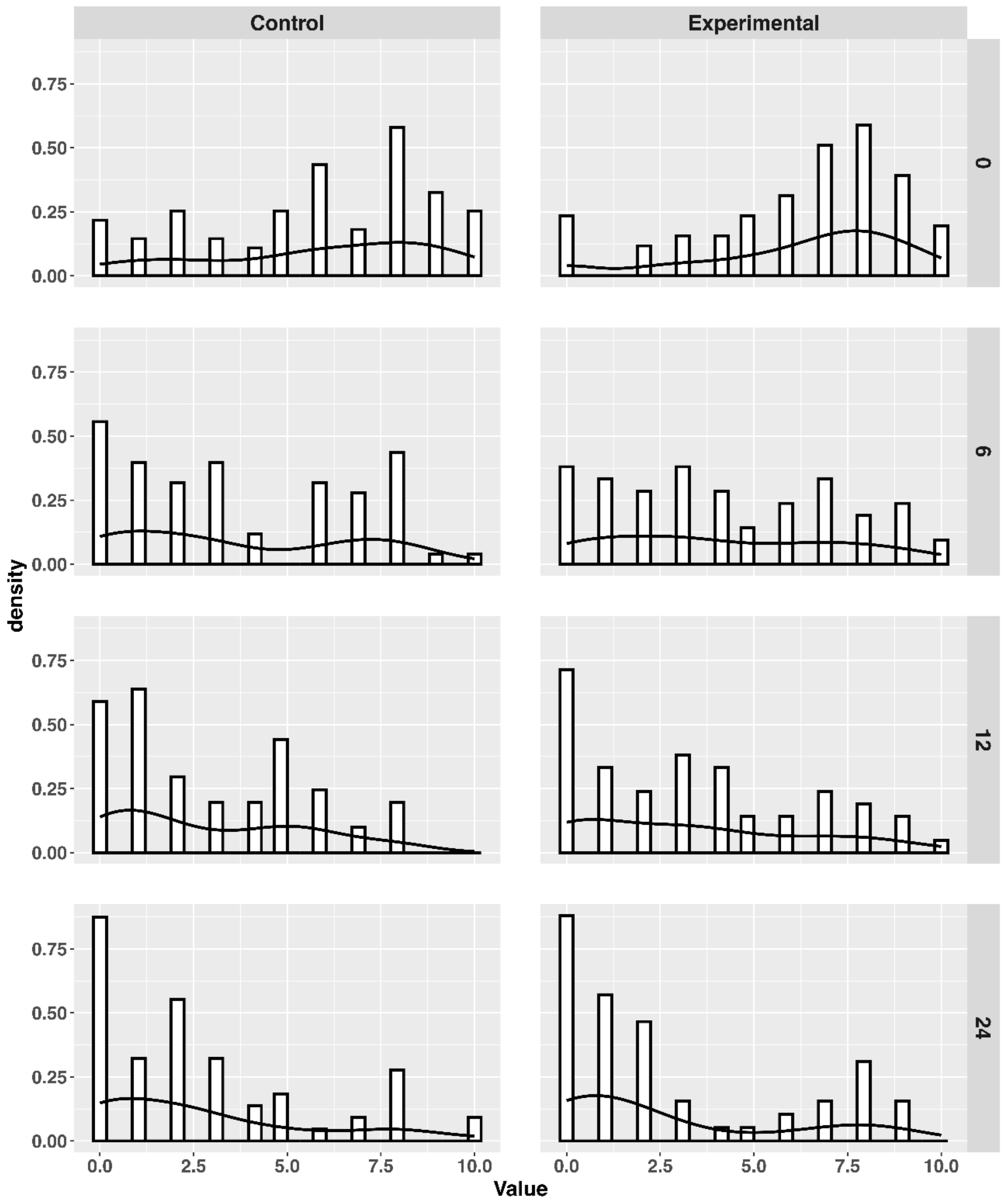

Figure S3. *Histograms of numeric pain rating scale at night.*

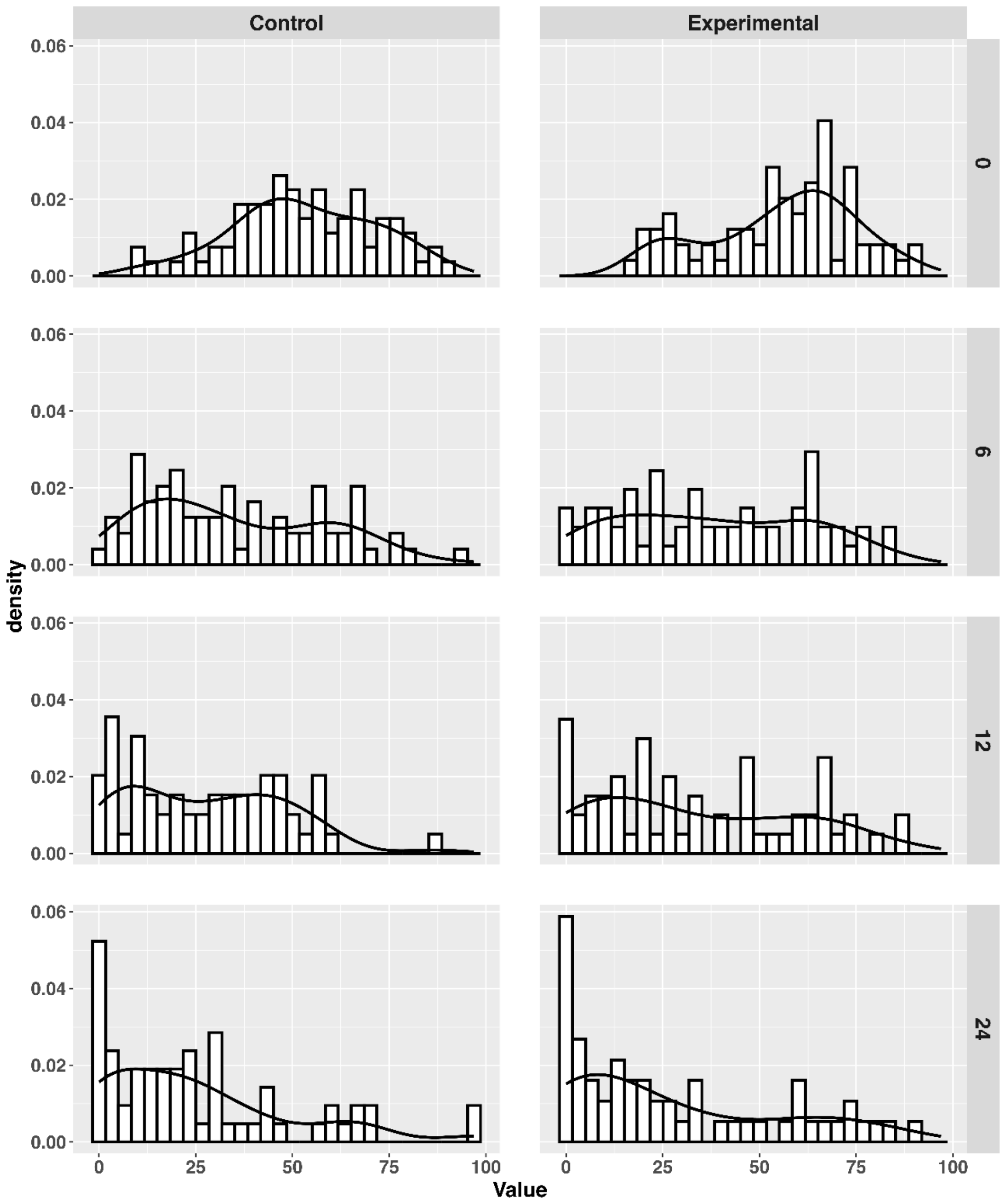

Figure S4. *Histograms of Shoulder Pain and Disability Index.*

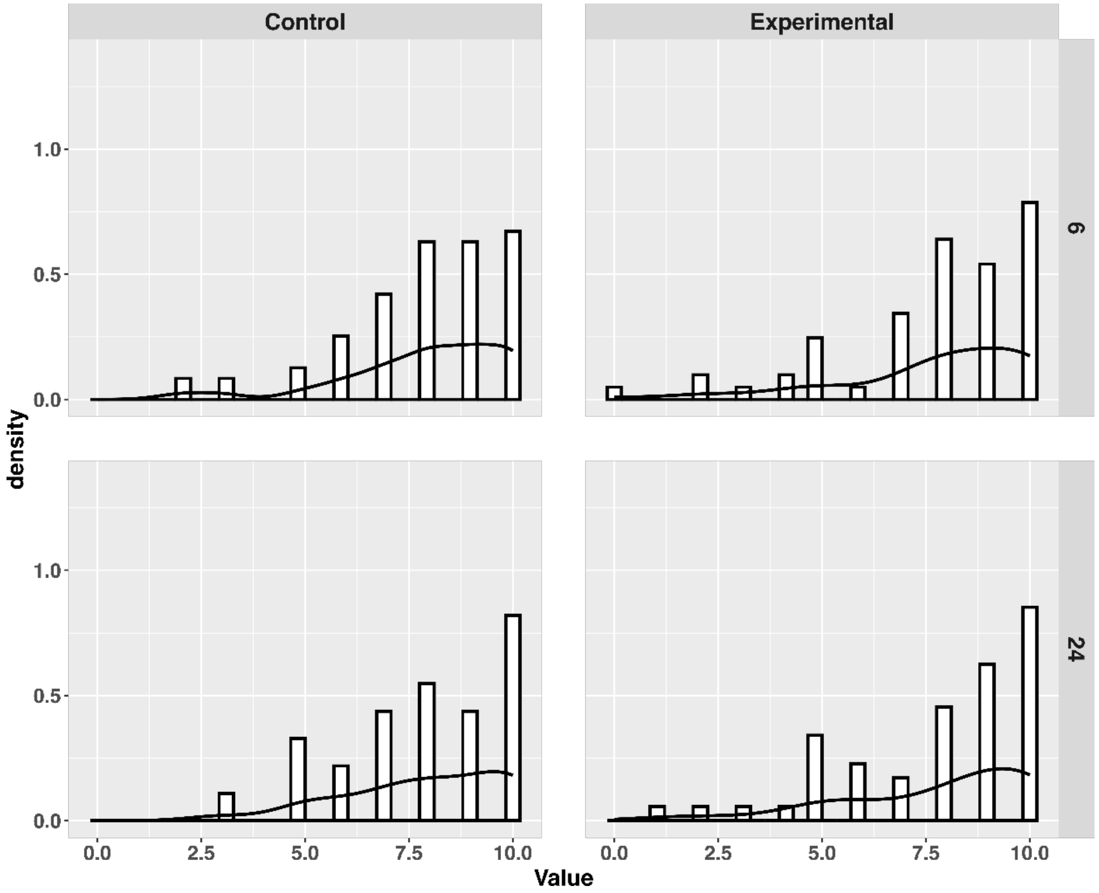

Figure S5. *Histograms of Patients' expectations.*
